# Optical Coherence Tomography in Chronic Relapsing Inflammatory Optic Neuropathy, Neuromyelitis Optica and Multiple Sclerosis: A Comparative Study

**DOI:** 10.3390/brainsci12091140

**Published:** 2022-08-27

**Authors:** Maziar Eslami, Samuel Lichtman-Mikol, Sara Razmjou, Evanthia Bernitsas

**Affiliations:** 1 Department of Neurology, University Health Center, Detroit, MI 48201, USA; 2 School of Medicine, Wayne State University, Detroit, MI 48201, USA; 3 Wayne State Neuroimaging Center, Sastry Foundation Neuro-Imaging Initiative, University Health Center, 8D, 4201 St. Antoine St., Detroit, MI 48201, USA

**Keywords:** optical coherence tomography, chronic relapsing inflammatory optic neuropathy, neuromyelitis optica, relapsing-remitting multiple sclerosis

## Abstract

**Purpose:** To examine the optical coherence tomography (OCT) features of the retina in patients with chronic relapsing inflammatory optic neuropathy (CRION) and compare them with those of neuromyelitis optica spectrum disorder (NMOSD), relapsing-remitting multiple sclerosis (RRMS) with and without optic neuritis (ON), and healthy controls (HC). **Methods:** In this retrospective cross-sectional study, we used spectral domain OCT to evaluate the retinal structure of 14 participants with CRION, 22 with NMOSD, 40 with RRMS with unilateral ON, and 20 HC. The peripapillary retinal nerve fiber layer (pRNFL), total macular volume (TMV), and papillomacular bundle (PMB) were measured, and intra-retinal segmentation was performed to obtain the retinal nerve fiber (RNFL), ganglion cell (GCL), inner plexiform (IPL), inner nuclear (INL), outer plexiform (OPL) and outer nuclear (ONL) layer volumes. **Results:** The global pRNFL [39.33(±1.8) µm] and all its quadrants are significantly thinner in CRION compared with all other groups (*p* < 0.05). CRION patients have decreased volumes of TMV, RNFL, GCL, and IPL compared with all other groups (*p* < 0.05). **Conclusion:** Severe thinning in pRNFL and thinning in intra-retinal segments of IPL, GCL, RNFL, and TMV could be helpful in differentiating CRION from NMOSD and RRMS.

## 1. Introduction

Chronic relapsing inflammatory optic neuropathy (CRION) is a steroid-responsive form of recurrent optic neuritis (ON) that was first described in 2003. Patients typically present with pain associated with visual loss. In most of the cases, the pain is severe and persistent, arising before or coincidentally with visual loss [1].

CRION is a rare disease that mainly affects patients between 30–50 years old. Relapses that occur after reducing or stopping steroids and a more severe degree of visual loss than in demyelinating optic neuritis are characteristic features [1,2]. The diagnostic criteria for CRION include at least one episode of ON, abnormal T2 lesion or enhancement contrast in the optic nerve, and steroid dependency with an excellent response to steroids. In 2014, Petzold and colleagues updated the diagnostic criteria for CRION and added seronegativity for AQP4-IgG [2]. Early recognition is very crucial, considering the risks of severe visual loss. Corticosteroids are used for acute attacks, while different other modalities, such as immunosuppressive medications, are used off-label as maintenance therapy. Although diagnostic criteria for the disease have been defined, CRION essentially remains a diagnosis of exclusion, as other demyelinating, autoimmune, and systemic causes should be ruled out first [1,2,3,4,5]. Despite the distinct clinical features characterizing CRION and NMOSD, such as a younger age of onset in CRION and female predominance in NMOSD, diagnosis remains challenging [5]. CRION patients may be incorrectly diagnosed with relapsing-remitting multiple sclerosis (RRMS), neuromyelitis optica spectrum disorder (NMOSD), or multiple sclerosis (MS)-mimics. Misdiagnosis can lead to mistreatment, causing potentially serious adverse events and unnecessary financial burden to the patient and society. In the context of similar symptomatology, clinical characteristics and paraclinical tests, such as lumbar puncture, serum biomarkers, and optical coherence tomography (OCT) may be helpful to establish the correct diagnosis [6,7]. Lumbar puncture is an invasive procedure that patients are usually unwilling to undergo. It can provide diagnostic evidence, but the findings are usually non-specific. Serum biomarkers, such as AQP4-IgG and MOG-IgG, although extremely helpful, might be associated with false negative or false positive results, especially in the early stages of the demyelinating disorder. Thus, there is an unmet need for a test that can be administered in a timely manner and provide the necessary information to avoid misdiagnosis.

OCT is a noninvasive, high-resolution method for the structural measurement of peripapillary retinal nerve fiber layer (pRNFL) thickness, optic nerve head volumetric analysis, and macular anatomy in patients with acute and chronic forms of optic neuropathies. It is a safe, reliable, and inexpensive technique for measuring the thickness of retinal layers to the level of microns [8]. This advanced technology allows for rapid visualization of the layers of the retina. RNFL is the innermost layer of the retina, consisting of unmyelinated axons originating from the ganglion cell neurons, which enters the neural retinal rim of the optic disc and, subsequently, coalesces as the optic nerve [8]. Retinal-layer thickness is used to monitor the progression of MS and the effect of modifiable risk factors for MS on retinal health and to mirror cortical atrophy in the MS population [9,10,11,12,13,14,15,16]. More recently, OCT outcomes have been used in clinical trials [16]. In this context, OCT might prove to be a valuable tool in differentiating between underlying diseases with overlapping features with the common presentation of ON. Our aim is to identify OCT differences between CRION-associated ON, NMOSD-associated ON, RRMS-associated ON, RRMS without ON, and healthy controls, which may be used in the differential diagnosis of these overlapping but different disease entities. 

## 2. Methods

**Figure 1:**
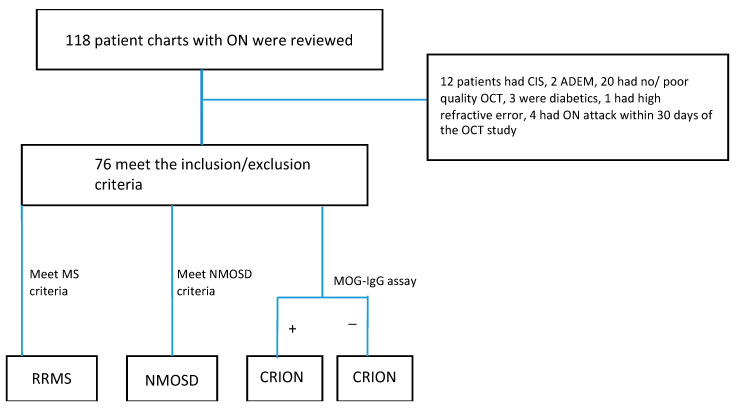
The flow chart depicts the steps of our study, from record identification to patient eligibility. It maps out the total number of records identified, the number of excluded cases and the reasons for exclusions, and the final number of eligible participants from each group. CIS: clinically isolated syndrome, ADEM: acute disseminated encephalomyelitis, RRMS: relapsing-remitting multiple sclerosis, CRION: chronic recurrent inflammatory optic neuropathy.

This is a retrospective, single-institution, cross-sectional study of 76 patients who were seen in our neuroimmunology clinic between 2016–2019. We assessed OCT features in 14 patients with CRION (5 patients had bilateral optic neuritis) and compared them with 22 patients with NMO (6 had bilateral optic neuritis), 40 patients with RRMS and history of unilateral ON, and 20 HC. The 40 patients with RRMS were then divided into 2 groups: 40 eyes affected by ON, and 40 eyes unaffected by ON. MS participants met the 2017 revised diagnostic criteria for MS, NMOSD participants met the 2015 updated diagnostic criteria for NMOSD, and CRION participants met the 2014 updated diagnostic criteria for CRION [2,17,18] {Figure 1}. All patients diagnosed with CRION had more than 1 episode of ON and underwent brain and spinal cord imaging that was normal. Patients were excluded if they had an attack of ON within the last 30 days before screening. In addition, patients with macular edema, glaucoma, and high refractive error >±6 diopters were excluded. Information about demographics, aquaporin-4 (AQP-4), and myelin oligodendrocyte glycoprotein (MOG) antibody status were obtained. Sex, race, and age were matched when possible. The local Institutional Review Board approved the study and written informed consent was obtained from all participants.

### 2.1. Imaging Technique

A trained OCT technician obtained all images using a single Heidelberg SPECTRALIS SD-OCT with N-Site Analytics platform, software version 6.0 (Heidelberg Engineering, Inc. Heidelberg, Germany). We measured pRNFL, total macular volume (TMV), and papillomacular bundle (PMB); moreover, intra-retinal segmentation was performed to obtain retinal nerve fiber (RNFL), ganglion cell (GCL), inner plexiform (IPL), inner nuclear (INL), outer plexiform (OPL), and outer nuclear (ONL) in CRION, and all were compared to NMOSD, RRMS with and without ON, and healthy controls (HC). 

To evaluate the thickness of the pRNFL, a single-line circular B-scan with a radius of 3.4 mm from the center of the papilla was used, with average automatic real time (ART) of 80. The TMV was measured as the volume between the inner limiting membrane and the boundary of the retinal pigment epithelium, within a 6 mm diameter circle centered on the fovea, as defined by the Early Treatment Diabetic Retinopathy Study (ETDRS) grid. A system built-in macular scan was used (30 20 mm^2^ area consisting of 61 B-scans, average ART of 9). All macular scans were subjected to automated segmentation using Heidelberg Engineering software, version 6.0. Final individual volume layer thicknesses were also measured within the ETDRS 6 mm circle between their respective boundaries. The following layers were automatically segmented: RNFL, GCL, IPL, INL, OPL, and ONL. Layers were not combined. All images were assessed for quality purposes, including evaluation for misalignments to avoid potential segmentation errors as outlined in the OSCAR IB criteria [19]. Quantitative OCT study results were reported according to the updated consensus APOSTEL recommendations [20].

### 2.2. Statistical Analysis

Statistical analysis was performed using the Statistical Package for Social Sciences, version 27 for Windows (SPSS^®^ Inc., Chicago, IL, USA). Demographics were compared between groups with one-way analysis of variance (ANOVA) and chi-squared test, as appropriate. Since there were cases where both eyes of a participant were included in the analysis, mean retinal thickness and volumes between CRION and the other diagnostic groups were compared using the linear generalized estimating equation (GEE) model to account for within-subject inter-eye correlation. *p*-values less than 0.05 are considered statistically significant. 

## 3. Results

### 3.1. Demographics

A total of 167 eyes from 96 participants were evaluated, including 19 eyes with CRION, 80 eyes with RRMS (40 with and 40 without Optic Neuritis), 28 eyes with NMO, and 40 eyes of HC. Ten CRION patients were AQP4-IgG negative and MOG-IgG positive; four were AQP4-IgG and MOG-IgG negative. All NMO patients were AQP4-IgG positive and MOG-IgG negative. The total average number of ON relapses in the CRION group was 3.2, the NMOSD group was 2.9, and the MS with ON was 2.5. The mean age was 42.8 for CRION participants, 39.6 for NMOSD, 37.5 for RRMS, and 36.4 for HC. In the CRION group, 8 patients (57.1%) were women, compared to 17 women (77.27%) in the NMOSD group, 30 (75%) in the RRMS, and 11 (55%) in HC. Five CRION (35.7%) patients had bilateral ON compared with six NMOSD (27.27%) and none RRMS participants. Nine participants were white (64.2%) in the CRION group compared with twelve white (54.5%) in the NMOSD group, twenty-three (57.5%) in the RRMS group, and ten white (50%) in the HC group. All participants were treated with disease modifying agents. Six (42.85%) CRION patients were treated with intravenous immunoglobulin, four (28.6%) with mycophenolate mofetil, and four (28.6%) with Rituximab. Thirteen (59.1%) NMOSD patients were treated with Rituximab, six (27.2%) with mycophenolate mofetil, and three (13.6%) with azathioprine. Twenty RRMS patients were on fingolimod (50%) and twenty on glatiramer acetate (50%). Clinical features, demographics, and treatment options for CRION patients are all presented in Table 1. 

### 3.2. Peripapillary RNFL (p-RNFL) Thickness in CRION vs. NMOSD

**Figure 2:**
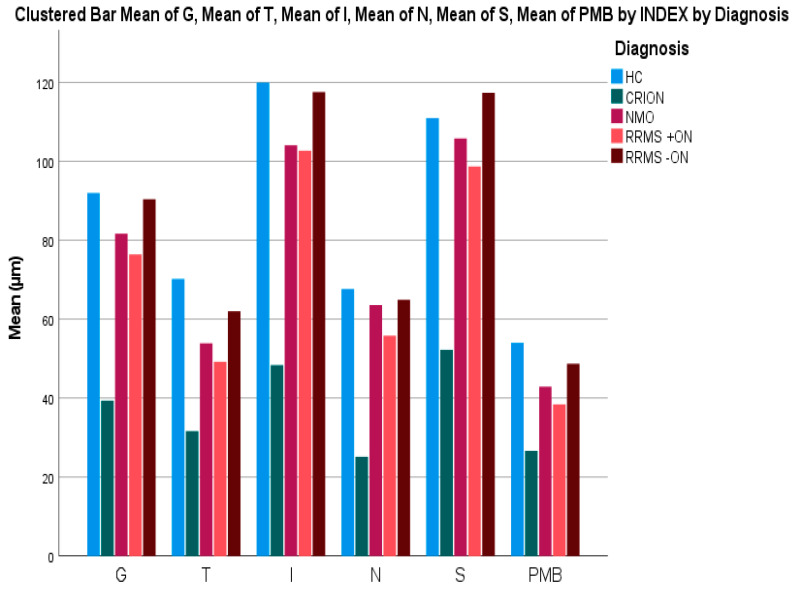
Comparison of all quadrants of pRNFL and PMB thickness between CRION, NMOSD, RRMS with ON, and RRMS without ON, and HC.

**Figure 3:**
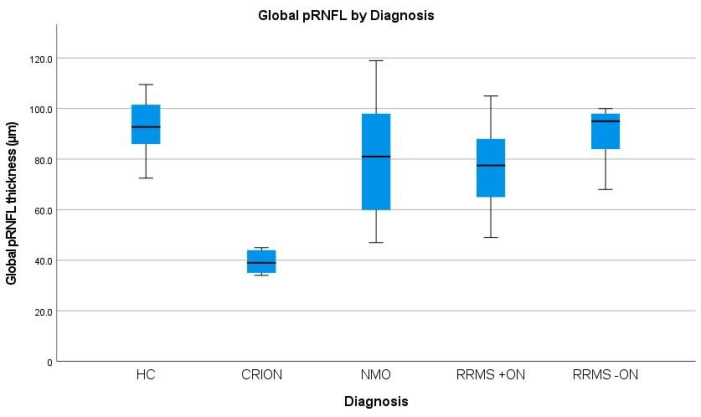
Comparison of global pRNFL in CRION, NMOSD, RRMS with ON (RRMS+ON), RRMS without ON (RRMS-ON), and healthy controls (HC).

CRION patients had significantly thinner global pRNFL (39.33 ± 1.8) than NMOSD participants (81.67 ± 7.7, *p* < 0.001). In addition, the average thickness of all quadrants (T, I, N, S) of CRION patients (31.67 ± 2.4, 48.33 ± 4.7, 25.17 ± 3.5, and 52.25 ± 5.1, respectively) was significantly lower compared to NMOSD (53.89 ± 6.25, 104.11 ± 9.86, 63.56 ± 5.92, and 105 ± 12.01, respectively; *p* = 0.016, *p* < 0.001, *p* = 0.001, and *p* = 0.004, respectively). To differentiate CRION from NMOSD, the global pRNFL and its inferior quadrant were the most sensitive (*p* < 0.001 and *p* < 0.001, respectively). Global *p*-RNFL and all quadrants results and comparisons between CRION and NMOSD are included in Table 2 and Table 3 and Figure 2 and Figure 3.

### 3.3. Intra-Retinal Segmentation in CRION vs. NMOSD

Retinal segmentation showed significantly thinner RNFL, GCL, IPL, TMV, and PBP in CRION (0.45 ± 0.01, 0.56 ± 0.03, 0.62 ± 0.02, 7.5 ± 0.09, and 26.67 ± 2.2, respectively) compared with NMO (0.70 ± 0.07, 0.87 ± 0.07, 0.77 ± 0.05, 8.09 ± 0.19, and 42.89 ± 4.30, respectively), with *p* = 0.014, *p* = 0.005, *p* = 0.031, *p* = 0.035, and *p* = 0.013, respectively. There was a non-significant trend for thicker ONL and INL in CRION compared to NMOSD (Table 2 and Table 3).

### 3.4. Peripapillary RNFL (p-RNFL) Thickness in CRION vs. RRMS±ON and HC

Mean and all quadrant thickness of pRNFL in CRION eyes was significantly reduced compared with RRMS with and without ON and HC. To differentiate CRION from RRMS+ON, the global pRNFL along with its superior and inferior segments were the most helpful (*p* < 0.001, *p* < 0.001 and *p* < 0.001 respectively) (Table 2 and Table 3 and Figure 2 and Figure 3). 

### 3.5. Intra-Retinal Segmentation in CRION vs. RRMS±ON and HC 

Intra-retinal segmentation showed significantly thinner TMV, RNFL, GCL, and IPL in CRION compared with RRMS + ON, with GCL being the most significant (*p* < 0.001). Similar results, with the addition of PMB being significant, were observed when compared CRION to RRMS without ON and CRION to HC (Table 2 and Table 3).

### 3.6. Intra-Retinal Segmentation and p-RNFL Thickness in NMO vs. RRMS±ON and HC

Comparison between NMOSD and RRMS + ON did not reach significance. However, global *p*-RNFL and its S, I, and T quadrant thicknesses were significantly reduced in NMOSD compared with RRMS-ON (*p* < 0.05). After intra-retinal segmentation, the GCL, IPL, and RNFL thicknesses of NMOSD patients reached significance when compared with RRMS-ON. As expected, pRNFL and all quadrant thicknesses were significantly higher in HC compared with NMOSD. Similarly, TMV, RNFL, GCL, IPL, and PMB were significantly higher in HC vs. NMOSD.

## 4. Discussion

We studied the OCT features in CRION, and we compared them with the most commonly seen CRION-mimics in clinical practice, such as NMOSD, RRMS with and without ON, and healthy controls. The participant groups were well-matched, with non-significant differences in age, gender, disease duration, and number of relapses. At the time of their recruitment, all participants met the most updated diagnostic criteria for their respective disease, and no changes in diagnosis were made in a 5-year follow-up period. Consistent with previous reports that showed a robust association of CRION with MOG-IgG status, the majority (75%) of our CRION patients were MOGA-IgG seropositive [21,22]. 

We demonstrated that both the global pRNFL layer as well as the temporal, inferior, nasal, and superior quadrants in CRION patients were significantly thinner than those in all other groups. Furthermore, the TMV, PMB, RNFL, GCL, and IPL volumes were significantly lower in CRION than NMOSD, RRMS±ON, and HC.

There is a paucity of the relevant literature, with a very limited number of studies that compare more than two groups of different-etiology ON-affected patients. Moreover, most of the studies showed inconsistent results, especially when comparing the retinal layer thickness between MOG-IgG+ON and AQP4-IgG+ON patient groups. Bichuetti and colleagues compared only the pRNFL thickness between CRION, NMOSD, and RRMS patients [23]. The investigators concluded that changes in pRNFL thickness are similar between CRION and NMOSD but distinct from RRMS. No reports of the MOGA status of the participants were available in this study. A second study compared four arms, including AQP4-IgG+ NMOSD, MOG-IgG+ ON, idiopathic ON, and MS. The study focused on pRNFL and GCIPL in these four groups and demonstrated a significant decrease in pRNFL and its superior and inferior quadrant thicknesses in the first three groups, relative to those of MS-associated ON. However, it failed to demonstrate any significant difference in pRNFL or GCIPL thicknesses between AQP4-IgG+ON and MOG-IgG+ON [24]. 

In a recent retrospective study, Chen JJ and colleagues included participants with MS-associated acute ON and MOG-IgG+ON; both groups were screened at the time of the acute ON attack. After comparing the pRNFL thickness between the groups, the investigators demonstrated that acute ON eyes from MOG patients had significantly thinner pRNFL compared with the acute ON eyes from MS patients. A RNFL cutoff of 118 µm was proposed, which provided a sensitivity of 74% and specificity of 82% in differentiating MOG from MS. The study included 64 MOG-IgG+ patients, a number high enough to allow evaluation of the RNFL performance, using receiver operating characteristic curves [25]. In a pediatric cohort including children with acute ON, Chen Q and colleagues followed-up MOG-IgG+ON, AQP4-IgG+ON, and double-seronegative ON patients for 6 months, after one episode of acute ON, and reported a trend for thinner global pRNFL in MOG-IgG+ON compared with AQP4-IgG+ON; however, no statistical significance was reached. Moreover, in both MOG-IgG+ON and AQP4-IgG+ON, the global pRNFL was significantly thinner compared with the double-seronegative ON [26]. On the contrary, in a single-institution study targeting a small and diverse cohort, Havla and colleagues reported significantly lower global pRNFL thickness in MOG-IgG+ON compared with AQP4-IgG+ON and MS-associated ON, while the non-ON eyes in MOG-IgG+ patients had significantly thinner T quadrants compared with the non-ON eyes of the MS and NMOSD patients [27]. 

In our study, we took these results one step further, and we studied all pRNFL quadrants and performed intra-retinal segmentation in four groups of patients and an additional group of HC. We have not examined OCT changes during an episode of an acute ON, as our participants underwent their OCT examinations more than 30 days after an acute attack. Although we concur with the more severe damage of pRNFL in CRION compared to RRMS, we were not able to reproduce the lack of significant difference between NMOSD and CRION observed in some of the studies [28]. Further analysis of our data demonstrated more severe pRNFL thinning in all quadrants in CRION than in both NMOSD and RRMS with and without ON. The low number of CRION and NMOSD participants did not allow us to evaluate the performance of pRNFL and its inferior quadrant in distinguishing CRION from NMOSD. 

After performing intra-retinal segmentation, we demonstrated significant thinning in RNFL, GCL, IPL, and TMV in CRION patients compared to all other groups. Interestingly, we observed a trend for thicker ONL and INL in CRION compared to NMO, RRMS with and without ON, and HC. Several previous studies reported increased nuclear-layer thickness after acute ON. Kaushik and colleagues described INL thickness as inversely proportional to retinal ganglion cell loss in optic neuritis [29]. More recently, Al-lousi and colleagues demonstrated the transient thickness of the nuclear layers, mainly in ONL+PS and to a lesser degree in OPL+INL, after one episode of acute ON, which is directly proportional to GCIPL thinness. The thickness reached its maximum at 4 months and declined thereafter; however, it remained elevated for 12 months compared to baseline [30].

Although we did not demonstrate significant differences between NMOSD and RRMS+ON, we found significant differences between NMOSD and RRMS without ON, NMOSD and HC, and RRMS±ON and HC. The lack of significance between NMOSD and RRMS+ON might be related to the high number of ON attacks observed in our RRMS+ON group in addition to the higher disease duration in the MS cohort that contribute to subclinical loss of retinal layer thickness. The significant greater thickness loss in all disease groups compared with HC was expected and in line with previous reports [28,31,32].

Limitations of our study include a small sample size, especially for the CRION patients, a single-center study with a retrospective, and a cross-sectional design that cannot prove causation. In addition, all study patients including CRION, NMOSD, and RRMS with and without ON were on different disease-modifying therapies. There are limited data on the effect of different immunomodulatory treatments on retinal integrity, and this could potentially skew our results, as we cannot eliminate the possibility of treatment heterogeneity with different impact on retinal injury [33]. Furthermore, we did not take into consideration various lifestyle factors, such as race, obesity, sleep duration and quality, and smoking that might interfere with the interpretation of our findings, as they can potentially affect the retinal structure and contribute to RNFL thinning [10,11,12]. Moreover, we have not studied any correlations with visual outcomes or serum neurofilament light chain levels that previous reports showed positive correlation with accelerated rates of neuro-axonal loss association in RRMS±ON [34]. These limitations necessitate cautious interpretation of our results and emphasize the need for longitudinal multicenter OCT studies on a larger number of patients to help shed more light on the OCT differences among these potentially disabling diseases.

In conclusion, our study showed severe pRNFL thinning in CRION, which is significant compared to NMOSD, RRMS with and without ON, and healthy controls. We observed decreased volumes in intra-retinal segments (RNFL, GCL, TMV, and IPL) in CRION that are significantly different from NMOSD and the rest of the groups. In addition, we found that ONL is thicker in CRION comparing to NMOSD and RRMS (with or without ON), but the difference did not reach statistical significance. We conclude that CRION has notable OCT features, such as severe thinning in pRNFL and decreased volumes in the intra-retinal segments of TMV, IPL, GCL, and RNFL. This may be helpful to distinguish CRION from NMOSD and RRMS, especially in atypical cases with overlapping features, but further research is needed to increase our knowledge about the OCT characteristics in such a rare disease. Future research should target a larger diverse sample with a larger proportion of CRION participants, which will provide higher statistical power and allow for more complex statistical analysis. Differentiating between CRION MOG-IgG positive and CRION MOG-IgG negative, as well as longitudinal OCT data in our study groups, will be of interest. Multicenter, collaborative trials, given the rarity of CRION, may answer questions and improve our understanding of these inflammatory eye-affecting diseases. 

## Figures and Tables

**Table 1:** Demographics and clinical characteristics of study participants.

	CRION (*n* = 14)	NMOSD (*n* = 22)	RRMS (*n* = 40)	HC (*n* = 20)
Age (years) ± SD	42.8 ± 8.5	39.6 ± 7.2	37.5 ± 5.9	36.4 ± 5.7
Sex (W/M)	8/6	17/5	30/10	11/9
Race (Wh/B)	9/5	12/10	23/17	10/10
Disease duration ± SD	3.5 ± 2.1	4.2 ± 3.8	5.6 ± 4.1	NA
B/l ON (*n*, %)	5 (37.5%)	6 (27.27%)	none	NA
AQP4-IgG (+/−)	0/14	22/0	0/40	NA
MOG-IgG (+/−)	10/4	0/22	0/40	NA
Relapses ± SD	3.2 ± 0.8	2.9 ± 1.1	2.5 ± 2.1	NA
Disease-modifying treatment	IVIg: 6 (42.85%) MM: 4 (28.6%) Rituxan: 4 (28.6%)	AZA: 3(13.6%) MM: 6 (27.2%) Rituxan:13(59.1%)	GA: 20 (50%) Fingo: 20 (50%)	

N: number, SD: standard deviation, CRION: chronic relapsing inflammatory optic neuropathy, NMO: neuromyelitis optica, RRMS: relapsing-remitting multiple sclerosis, HC: healthy controls, W/M: women/men, Wh/B: white/black, AQP4: aquaporin 4. MOG: myelin oligodendrocyte glycoprotein, b/l: bilateral, NA: not available, IVIg: intravenous immunoglobulin, MM: mycophenolate mofetil, Rituxan: rituximab, AZA: azathioprine, GA: glatiramer acetate, Fingo: flingolimod.

**Table 2:** OCT Data of all participants (CRION, NMOSD, RRMS+ON, RRMS-ON, and HC).

	CRION	NMOSD	RRMS+ON	RRMS-ON	HC
**Peripapillary**					
**RNFL**					
pRNFL (µm)	39.33 (±1.8)	81.67 (±7.7)	86.40 (±5.1)	90.40 (±3.2)	92.00 (±3.5)
T (µm)	31.67 (±2.4)	48.89 (±6.3)	49.20 (±5.4)	62.00 (±4.2)	70.20 (±3.6)
I (µm)	48.33 (±4.7)	100.11 (±9.9)	102.7 (±5.9)	117.6 (±4.7)	120.0 (±5.8)
N (µm)	25.17 (±3.5)	63.56 (±5.9)	55.8 (±4.6)	64.90 (±2.6)	67.65 (±4.7)
S (µm)	52.25 (±5.1)	105.83 (±12.0)	115.7 (±7.0)	117.4 (±5.5)	111.0 (±4.6)
**Intra-retinal**					
**Segmentation**					
TMV (mm^3^)	7.5 (±0.09)	8.1 (±0.19)	8.25 (±0.0.16)	8.47 (±0.13)	8.55 (±0.11)
RNFL (mm^3^)	0.45 (±0.01)	0.70 (±0.07)	0.70 (±0.06)	0.83 (±0.04)	0.92 (±0.04)
GCL (mm^3^)	0.56 (±0.03)	0.87 (±0.07)	0.85 (±0.05)	1.00 (±0.04)	1.03 (±0.03)
IPL (mm^3^)	0.62 (±0.02)	0.77 (±0.05)	0.77 (±0.03)	0.86 (±0.03)	0.86 (±0.02)
INL (mm^3^)	1.03 (±0.03)	1.00 (0.02)	1.01 (±0.02)	1.00 (±0.02)	0.96 (±0.01)
OPL (mm^3^)	0.77 (±0.01)	0.77 (±0.01)	0.80 (±0.02)	0.81 (±0.02)	0.79 (±0.01)
ONL (mm^3^)	1.85 (±0.06)	1.75 (±0.05)	1.77 (±0.06)	1.75 (±0.06)	1.79 (±0.06)
PMB (µm)	26.67 (±2.2)	42.89 (±4.3)	38.40 (±4.3)	48.70 (±3.1)	54.00 (±2.3)

Aggregate statistics are reported as mean (±standard error of mean). Abbreviations: optical coherence tomography (OCT), healthy controls (HC), chronic relapsing inflammatory optic neuropathy (CRION), neuromyelitis optica spectrum disorder (NMOSD), relapsing-remitting multiple sclerosis with optic neuritis (RRMS+ON), relapsing-remitting multiple sclerosis without optic neuritis (RRMS-ON), temporal (T), inferior (I), nasal (N), superior (S), total macular volume (TMV), papillomacular bundle (PMB), peripapillary retinal nerve fiber layer (pRNFL), retinal nerve fiber layer (RNFL), ganglion cell (GCL), inner plexiform (IPL), inner nuclear (INL), outer plexiform (OPL), outer nuclear (ONL) layers, non-significant (NS).

**Table 3:** OCT data comparison between CRION and NMOSD, CRION and RRMS+ON, CRION and RRMS-ON, CRION and HC (GEE).

	*p*-Value	*p*-Value	*p*-Value	*p*-Value
**Peripapillary RNFL**	CRION vs. NMOSD	CRION vs. RRMS +ON	CRION vs. RRMS -ON	CRION vs. HC
pRNFL (µm)	<0.001	<0.001	<0.001	<0.001
T (µm)	0.016	0.031	<0.001	<0.001
I (µm)	<0.001	<0.001	<0.001	<0.001
N (µm)	0.001	0.001	<0.001	<0.001
S (µm)	0.004	<0.001	<0.001	<0.001
**Intra-Retinal Segmentation**				
TMV (mm^3^)	0.035	0.005	<0.001	<0.001
RNFL (mm^3^)	0.014	0.005	<0.001	<0.001
GCL (mm^3^)	0.005	<0.001	<0.001	<0.001
IPL (mm^3^)	0.031	0.005	<0.001	<0.001
INL (mm^3^)	0.560	0.773	0.479	0.060
OPL (mm^3^)	0.820	0.272	0.270	0.695
ONL (mm^3^)	0.242	0.419	0.329	0.591
PMB (µm)	0.013	0.066	<0.001	<0.001

Optical coherence tomography (OCT), healthy controls (HC), chronic relapsing inflammatory optic neuropathy (CRION), neuromyelitis optica spectrum disorder (NMOSD), relapsing-remitting multiple sclerosis with optic neuritis (RRMS+ON), relapsing-remitting multiple sclerosis without optic neuritis (RRMS-ON), temporal (T), inferior (I), nasal (N), superior (S), total macular volume (TMV), papillomacular bundle (PMB), peripapillary retinal nerve fiber layer (pRNFL), retinal nerve fiber layer (RNFL), ganglion cell (GCL), inner plexiform (IPL), inner nuclear (INL), outer plexiform (OPL), outer nuclear (ONL) layers, non-significant (NS).

## Data Availability

Data will be available after contacting the senior author (E.B.).
